# Nutraceuticals and Metabolic Health: Can Supplements Prevent Metabolic Syndrome? A Narrative Review

**DOI:** 10.3390/nu18213672

**Published:** 2026-11-05

**Authors:** Andrew J. Krentz, Oleksii Kozhevnikov, Jennifer E. McCallum, Lylah Drummond-Clarke, Megan Fant, Jing Han Ong, Sital Harris, Steven Walker

**Affiliations:** 1 School of Life Course & Population Sciences, King’s College London, Strand, London WC2R 2LS, UK; 2 MedibiotiX GmbH, Dr.-Reckeweg-Str. 2-4, 76532 Baden-Baden, Germany; oleksii.kozhevnikov@medibiotix.net; 3 St Gilesmedical London & Berlin, Suite 616, The Shepherds Building, Charecroft Way, West Kensington, London W14 0EE, UK; jennifer.mccallum@stgmed.com (J.E.M.); lylahdc@stgmed.com (L.D.-C.); cadence.ong@stgmed.com (J.H.O.); steven.walker@stgmed.com (S.W.); 4 School of Life Sciences, University of Sussex, Brighton BN1 9RH, UK; 5 School of Life Sciences, University of Dundee, Carnelley Building, Dundee DD1 4HN, UK; megan.fant@stgmed.com; 6 Life Science Academy, Eastbourne, East Sussex BN21 3FG, UK; sital@responsiva.biz; 7 Medical School, University College London, 74 Huntley St, London WC1E 6DE, UK

**Keywords:** metabolic syndrome, MetS, health, supplements, nutraceuticals, prevention, cardiovascular disease, obesity, type 2 diabetes, risk factors, inflammation, insulin resistance, dyslipidaemia

## Abstract

Metabolic syndrome (MetS) encompasses a spectrum of disease pathologies that include dyslipidaemia, insulin resistance, hypertension, obesity, and inflammation. The rising prevalence of MetS and the associated morbidity and mortality represent a growing burden on global healthcare. MetS’s hallmarks are readily quantified and can be used to assess development and progression of the syndrome. Nutraceuticals, bioactive compounds derived from natural sources such as plants, fruits, cultures and grains, have a potential role in protection against cardiometabolic diseases. Relevant actions of nutraceuticals include their antioxidant, probiotic, anti-inflammatory, insulin-sensitising, lipid-modifying, anti-adiposity, and endothelial protective functions. This review explores the evidence for common nutrient deficiencies in metabolic syndrome alongside their role in preventing and modifying the complex elements that characterise this prevalent disease cluster.

## 1. Introduction

Metabolic syndrome (MetS) is a major health crisis affecting an estimated 1.54 billion adults worldwide [1]. First described by Reaven in 1988 as ‘Syndrome X’, it comprises a collection of pathologies including abdominal obesity, hypertension, insulin resistance (IR), and dyslipidaemia (Reaven did not include excess adiposity—now regarded as a core initiator and driver of metabolic syndrome—in his original report), which, in turn, are associated with considerable morbidity and premature mortality [2,3]. Individuals with MetS have double the risk of developing cardiovascular disease (CVD) within 5 to 10 years—a risk that continues to increase throughout the lifetime. MetS is also associated with a five-fold increased risk of type 2 diabetes (T2D) [4]. In addition to CVD and T2D, other associated comorbidities include Metabolic Dysfunction-Associated Steatotic Liver Disease (MASLD), polyendocrine metabolic ovarian syndrome (PMOS) (previously polycystic ovarian syndrome (PCOS)), obstructive sleep apnoea, hypogonadism, lipodystrophy, and microvascular disease [5]. The prevalence of MetS and its accompanying risks increase in tandem with age-related increases in obesity [5].

Obesity, particularly visceral adiposity, is central to the development of MetS [6]. Avoiding and reversing the deposition of adipose fat is key to preventing and managing MetS. The association between obesity and MetS was highlighted in a 2023 study that identified a 65% prevalence of MetS within a cohort of T2D patients [7]. The benefits of weight management in individuals with T2D are well documented, with the landmark DiRECT study achieving diabetes remission in 46% of those who partook of a low-energy formula diet intervention (825–853 kcal/day for 12–20 weeks) and in 86% of participants who lost 15 kg [3,8].

Increased obesity and its associated risks are an increasing public health concern and healthcare burden. For adults aged >18 years, the World Health Organization (WHO) defines overweight as a body mass index (BMI) of 25.00–29.99 kg/m^2^ and obesity as a BMI that is 30 kg/m^2^ [9,10]. Between 1990 and 2022, the number of overweight adults grew from 25% to 43%, and the prevalence of obesity more than doubled worldwide. This increase can, in part, be linked to increased consumption of ultra-processed foods in regions characterised by higher levels of socioeconomic inequity and lower intake of animal-sourced foods in high-income countries [11,12].

Currently, first line, non-pharmacological defences against obesity, MetS, CVD, and T2D centre on healthy nutrition—specifically, diets that prioritise fruits, vegetables, whole grains, legumes, healthy fats, and regular physical activity in the context of appropriate caloric intake [11,13]. Castro-Barquero et al. conducted a review comparing dietary patterns and their effect on MetS and its individual components [14,15,16]. All of the studied approaches (Mediterranean, DASH, plant-based, low-carbohydrate, low-fat, high-protein, and Nordic diets, as well as intermittent fasting) had positive effects, highlighting the benefits of healthy dietary changes rather than restricting one food group or macronutrient [16]. Here, the term ‘nutrient’ refers to essential chemical compounds naturally found in food. Though effective, individuals’ adherence to a recommended diet can be challenged in part by economic realities; this is unlikely to improve, given the few existing policies that support accessibility of affordable, healthy foods [10]. Unsurprisingly, socioeconomically disadvantaged populations have experienced the greatest decline in metabolic health in recent years. Among at-risk individuals, planning and preparing healthy meals, along with meeting the recommended levels of habitual physical activity, require time and material resources that may not be practical or sustainable [10,17]. Even dietary approaches not limited by cost, such as intermittent fasting and intense caloric restriction, may fail without supervision or commitment [8,15]. Given these challenges, effective treatment strategies designed to reduce excess adiposity while promoting weight maintenance are necessary [18,19].

Recent advances in pharmacotherapeutics have revolutionised the management of cardiometabolic disease. Glucagon-like peptide-1 receptor agonists (GLP-1 RAs) have multiple benefits for components of MetS. Drugs in this class reduce CVD and T2D burden, suggesting a potential benefit across other indices of MetS. GLP-1 RAs improve insulin sensitivity; promote weight loss; and reduce blood pressure, lipid levels, and inflammation, all of which play a role in the pathophysiology of MetS [19,20,21]. Sandsal et al. investigated the effects of GLP-1 RA liraglutide alone or in combination with exercise on the severity of MetS after weight loss. When compared to placebo, the MetS severity z-score was reduced by 0.37 by liraglutide and 0.48 by liraglutide and exercise [19]. While these results are promising, GLP-1 RA treatments have some limitations, including lack of accessibility across healthcare systems, adverse effects, and the need for long-term treatment [22]. Moreover, a recent and comprehensive network meta-analysis comparing different GLP-1 RA formulations revealed that, despite significant weight loss, the drugs failed to influence individuals’ quality of life at one year [23]. By comparison, bariatric and metabolic surgeries are generally effective for weight loss and in reducing the prevalence of MetS and IR in the short and medium term [24]. However, such a major procedure is associated with a small risk of death (0.2%); serious complications (4%); and, in a minority, long-term gastro-intestinal and nutritional complications [25].

Emerging evidence suggests the gut microbiome may play a role in the development of MetS [26]. The intestinal microbiome consists of approximately 3.3 million unique genes and approximately 1000 different bacterial species. These bacteria are critical in regulating gut metabolism and supporting the host’s immune system [27]. Backhed et al. first demonstrated the association between the gut microbiome and obesity in mice. When compared to germ-free mice, mice transplanted with a conventional microbiota had a 60% increase in body fat, as well as significantly elevated fasting blood glucose (FBG), insulin levels, and IR [28]. In humans, the proportion of Bacteroidetes, a dominant group of beneficial gut bacteria, was found to be lower in obese individuals compared to lean individuals [29]. Moreover, a study that infused intestinal microbiota from healthy participants in participants with MetS observed increased levels of insulin sensitivity in the recipients [30]. A connection between microbiota and MetS has been reinforced by studies reporting improvements in metabolic health after probiotic supplementation with strains belonging to *Bifidobacterium animalis* and *Lactobacillus acidophilus* (*L. acidophilus*) families [31,32,33].

It has been suggested that dietary manipulation by means of the ingestion of certain nutraceuticals, such as probiotics, may be of benefit in preventing MetS [32,34,35]. Nutraceuticals, a portmanteau of “nutrition” and “pharmaceuticals”, are formulations of biologically active compounds that are developed with the aim of meeting specific dietary requirements and preventing or treating disease [36]. Increased consumer awareness, healthcare costs, and marketing support a rapidly expanding multi-billion-dollar nutraceutical industry [37]. However, their clinical use remains limited owing to a lack of awareness among healthcare professionals, safety concerns, and regulatory discrepancies [35]. The aim of this narrative review is to explore the landscape of nutraceuticals and their potential application in the prevention of MetS and its varied disease indices. We discuss evidence-based research exploring potentially valuable agents, as well as their adverse effects and safety, practical recommendations for their use, and future research needs.

## 2. Methods

This narrative review aimed to explore the value of nutraceutical interventions for preventative measures by examining reported outcomes (PICO) in populations at risk for metabolic syndrome. A comprehensive literature search was performed across PubMed, Embase, and Web of Science, using keywords relating to nutraceuticals and MetS. Preliminary search terms included ‘nutraceuticals’, ‘metabolic syndrome’, ‘obesity’, ‘-risk’, ‘type 2 diabetes’, ‘cardiovascular disease’, ‘endothelial dysfunction’, ‘hyperlipidaemia’, ‘gut dysbiosis’, ‘inflammation’, and ‘prevention’. Date restrictions were not applied, though studies published after 1989 were prioritised, given the term ‘nutraceutical’ was first introduced by Stephen DeFelice in 1989 [38]. Backward citation tracking was used to explore the reference lists of key articles generated through the search strategy, followed by forward citation tracking to identify novel research. Searches were supplemented by an exploration of grey literature (conference proceedings, theses, white papers, and government or NGO reports).

The sample included original research articles (randomised controlled trials, animal studies, observational studies, and experimental studies), cohort studies, clinical trials, meta-analyses, and systematic or narrative reviews that discussed the role of dietary components and nutraceuticals related to indices of MetS. Human clinical studies were prioritised while evidence derived from in vitro or in vivo research was utilised to illustrate proposed mechanisms of action (MOAs), where appropriate. Studies not published in English and involving those under 18 years of age were excluded. No formal risk-of-bias assessment or validated evidence-grading framework was undertaken. The strengths and limitations of the evidence summarised was appraised descriptively, considering the studied populations, trial design and sample size, as well as heterogeneity within included meta-analyses. Where available, GRADE certainty ratings reported in the included publications were also considered.

## 3. Pathophysiology of Metabolic Syndrome

**Figure 1:**
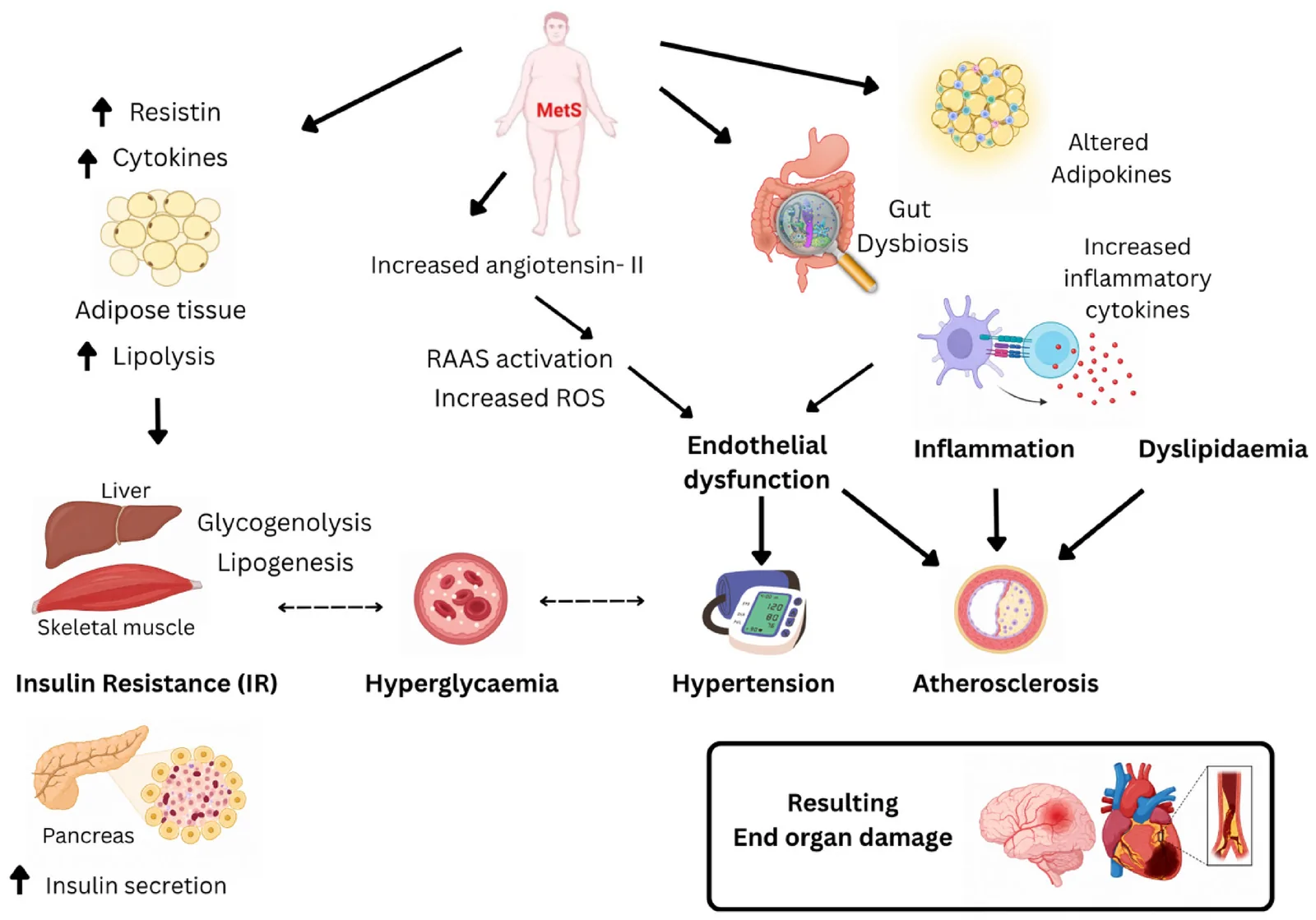
Summary of the pathophysiological changes involved in metabolic syndrome (MetS) (RAAS: Renin–Angiotensin–Aldosterone System; ROS: Reactive Oxygen Species). Flow arrows indicate physiological pathways; ↑ indicate upregulation; two headed arrows indicate bidirectional pathways.

Overnutrition, leading to excess visceral adipose tissue, is a demonstrated key trigger for many of the mechanisms involved in the pathophysiology of MetS. In addition to storing lipids, adipose tissue in obese humans overproduces various adipokines, including inflammatory cytokines, hormones, and peptides. The consequent IR, neurohormonal activation, and chronic inflammation are closely associated with the development and progression of MetS, CVD, and T2D, as summarised in Figure 1 [20].

Obesity is associated with a state of heightened innate immunity that increases the risk of inflammatory diseases, including T2D and atherosclerosis, often in the context of MetS [39,40]. This chronic state of inflammation is a result of the inflammatory cytokines (tumour necrosis factor alpha (TNF-α), interleukin-1 (IL-1), and interleukin-6 (IL-6)), hormones released by adipose tissue, and the activation of inflammatory signalling pathways driven by oxidative stress [41]. Overproduction of TNF-α contributes to IR and the development of MetS via phosphorylation and inactivation of insulin receptors, increasing the circulating levels of non-esterified fatty acids (NEFAs) by promoting adipocyte lipolysis and by inhibiting of the release of insulin-sensitising adiponectin [42,43]. Increased production of IL-6 leads to increased levels of C-reactive protein (CRP), which are associated with the development of MetS, T2D, and CVD [44]. In obesity, hormones released by adipocytes also contribute to inflammation. Elevated levels of leptin and visfatin, alongside decreased adiponectin levels, are also associated with an increased immune response [45].

In a healthy individual, insulin suppresses hepatic glucose production, increases glucose uptake in the liver and muscles and inhibits lipolysis and glucogenesis [46]. These actions have distinct organ- and pathway-specific dose–response relationships. When IR develops in response to obesity, the inhibition of lipolysis by insulin is impaired while re-esterification is reduced to release NEFA into the circulation. In muscle, NEFA decreases the uptake of glucose. In the liver, higher rates of NEFA delivery drive glucogenesis and lipogenesis. In order to maintain a normal blood glucose level, the pancreatic islet β-cells synthesise and secrete more insulin. However, in individuals predisposed to develop T2D, this state is not sustainable, and eventually, insulin secretion decreases [47]. Furthermore, NEFAs are lipotoxic to pancreatic β-cells and, thus, when present in excess, impair insulin secretion [39,48].

In the liver, an increase in circulating NEFAs alters lipid metabolism [46]. The concentration of high-density lipoprotein (HDL) in the circulation is decreased, and elevated NEFA levels increase triglyceride synthesis to increase the production of triglyceride-rich very-low-density lipoproteins (VLDLs) [49]. This pattern of dyslipidaemia is characteristic of MetS [20,39].

Hyperglycaemia and excess NEFA both cause an overproduction of reactive oxygen species (ROS), which activates nuclear factor κB (NFκB) and increases formation of advanced glycation end products (AGEs) [48,50]. Activation of NFkB promotes inflammation, and an increase in AGEs damages endothelial cells, leading to the production of inflammatory cytokines [39,48,50,51]. Furthermore, inflammatory cytokines, including those released by excess adipose tissue, promote ROS production and lipid peroxidation to worsen the inflammatory state [41].

Oxidative stress, due to excessive ROS production and reduced antioxidant activity, is associated with all of the pathophysiological components of MetS: obesity, dyslipidaemia, hyperglycaemia, IR, hyperinsulinemia, and hypertension [39]. Multiple pathways that cause oxidative stress contribute to the state of chronic inflammation present in MetS. Nitric oxide (NO) is a potent vasodilator produced by endothelial nitric oxide synthase (eNOS). In MetS, increased ROS production activates the protein kinase C (PKC) and hexosamine pathways, and these inhibit NO production via decreased eNOS production and inhibition of eNOS phosphorylation, respectively, together promoting the development of hypertension [39,48,52]. Additionally, obesity and IR are associated with increased production of endothelial vasoconstrictor angiotensin-II [52]. Angiotensin-II contributes to the increased production of ROS and promotes inflammation via its effects on PKC and hexosamine pathways [39,53].

### Gut Dysbiosis in Metabolic Syndrome

**Figure 2:**
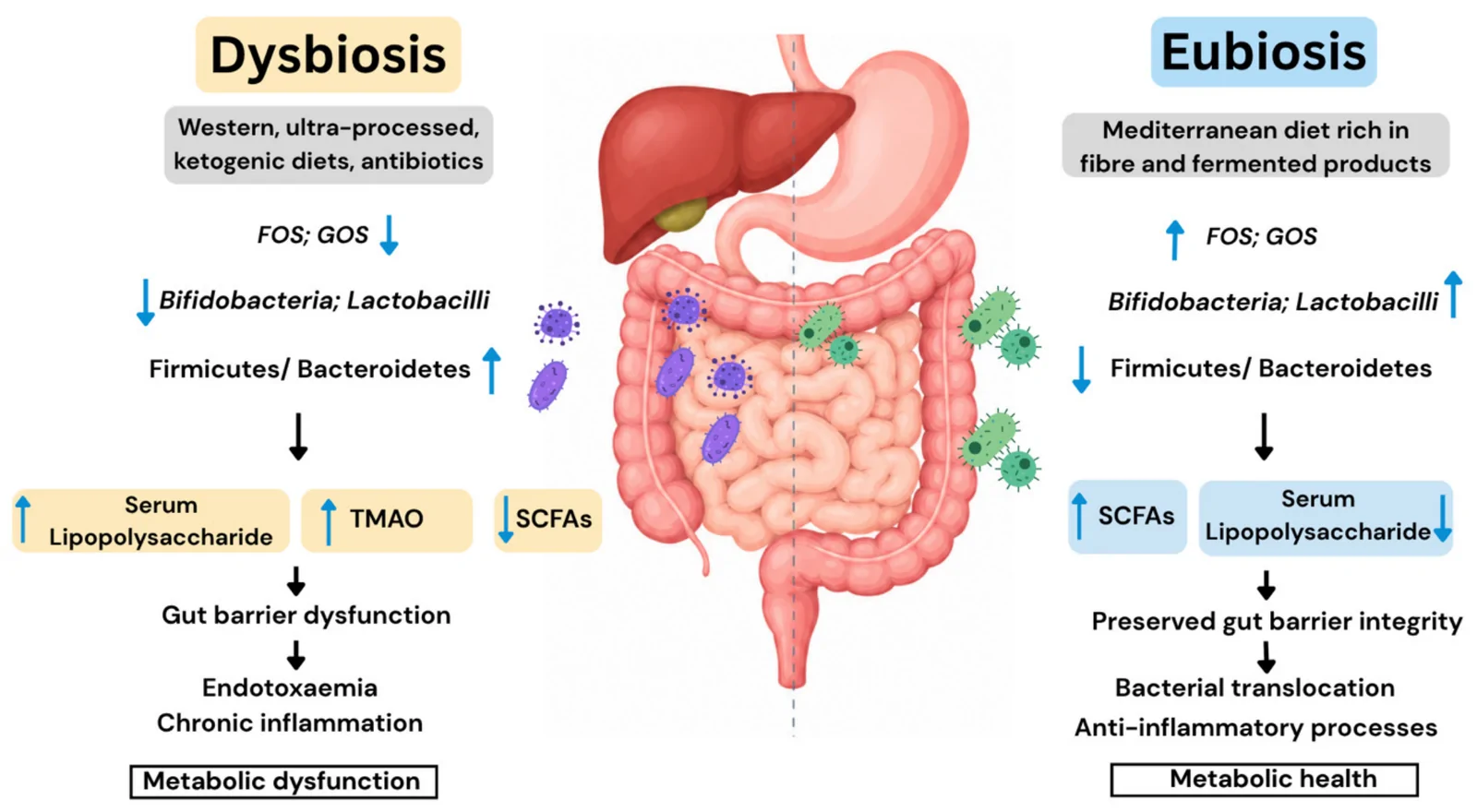
Comparison of gut dysbiosis and eubiosis. (Fructooligosaccharides (FOS), Galactooligosaccharides (GOS), Short Chain Fatty Acid (SCFA), Trimethylamine N-oxide (TMAO)). Flow arrows indicate physiological pathways in dysbiosis and eubiosis; ↑ indicate upregulation; ↓ indicate downregulation.

Gut dysbiosis (Figure 2) occurs when there is a disruption in the composition of the gut microbiota, characterised by reduced microbial diversity, loss of beneficial components, and expansion of opportunistic and pro-inflammatory taxa, as well as impaired microbial metabolic function [54,55]. Gut microbiota can be adversely affected by Western dietary patterns typically high in ultra-processed foods and low in fibre, probiotic foods, and variety. Antibiotics can also diminish gut microbial diversity, although they remain an essential tool in the effective treatment of bacterial infection [56]. Microbial changes can decrease production of small chain fatty acids (SCFAs) and increase intestinal permeability, thereby compromising intestinal barrier support and collectively contributing to low-grade immune activation [55]. Gut dysbiosis also alters microbial metabolic pathways that modulate the production of pro-inflammatory cytokines such as interferons (IFNs) and TNF [55]. This, in turn, can lead to chronic inflammation and metabolic dysfunction, both of which are hallmark features of MetS [55]. Evidence exploring the contributors to MetS development suggest gut dysbiosis may be a causal factor [57]. Efforts to better understand the relationship between the ‘gut–brain axis’, which proposes a bidirectional relationship, are also underway [58]. Hyperglycaemia and dyslipidaemia may disrupt the gut microbiota by altering the availability of resources—notably, bile acids and trimethylamine N-oxide (TMAO)—that govern microbial growth [59,60]. These findings suggest metabolic disturbances could perpetuate a feedback loop that exacerbates both MetS and dysbiosis, although the direction and causal nature of these relationships require further exploration.

## 4. Nutraceuticals and Metabolic Syndrome: The Evidence

### 4.1. Vitamin D

Fat-soluble vitamin D is an essential nutrient and hormone obtained from sunlight, dietary intake, and supplementation, with clinical evidence largely agreeing that adults and children should aim for a minimum concentration of 30 ng/mL [61]. While vitamin D is classically recognised for its role in calcium absorption and bone health [62,63], its role in preventing MetS is less well established. The status of vitamin D at baseline has been inversely associated with the prevalence of MetS and T2D in cross-sectional studies [64,65]. Observational data have linked lower 25(OH)D with markers of inflammation, IR and adiposity that accompany MetS, including higher IL-6 and hs-CRP, lower adiponectin, higher HOMA-IR, and greater BMI and waist circumference (WC) in Black and White adults [63]. Proposed mechanisms for the association of vitamin D deficiency with MetS include systemic inflammation, IR, and elevated blood pressure via activation of the renin–angiotensin–aldosterone system (RAAS) and inhibition of pancreatic β-cell function [66]. Correcting low vitamin D levels may seem plausible for preventing MetS, but the current evidence is sparse and inconsistent, with findings varying depending on the studied population.

In general populations, the available evidence does not show clear benefit for MetS. In a primary prevention setting, an ancillary study from the VITAL trial cohort involving 771 participants aged 50 and above found that two years of vitamin D3 at 2000 IU/day did not significantly change WC, a component of MetS, or other measures of adiposity and body composition, including weight, body mass index (BMI), total fat mass and lean tissue [67]. However, an exploratory finding revealed body fat percentage improved slightly in those with a normal BMI but not in overweight or obese participants (*p* for interaction = 0.04) [67]. In contrast, a smaller trial in 160 vitamin D-deficient postmenopausal women did report lower triglycerides, insulin and HOMA-IR and lower odds of MetS (OR 0.42, 95% CI 0.21 to 0.83) after nine months of 1000 IU/day [68]. Similarly, 2000 IU/day of vitamin D supplementation for 12 weeks in 60 PCOS women with vitamin D deficiency or insufficiency improved BMI, waist-to-hip ratio, insulin, HOMA-IR and lipids compared with diet and exercise alone, but only in women with obesity or insulin resistance [69]. Beyond vitamin D-deficient populations, supplementation may benefit glucose regulation in groups at risk. In people with prediabetes, pooled data from three RCTs suggest a modest reduction in progression to type 2 diabetes (adjusted HR of 0.85) [70]. Exploratory subgroup analyses point to other subgroup differences, with significant elevation of HDL-c in individuals with diabetes but higher LDL-c in younger adults and males [71].

Among individuals with MetS or obesity, trial findings are conflicting. Thirteen RCTs have shown lower fasting glucose, insulin resistance and blood pressure but no change in waist circumference, lipids or HbA1c [72], and another meta-analysis reported improved glycaemic control, insulin resistance and visceral fat [73]. However, others found no benefit [71,74], and certainty of evidence for effects on triglycerides and WC has been rated low to very low [75]. This variation may reflect differences in doses, formulation, treatment duration, study populations and baseline vitamin D status. In MASLD, vitamin D supplementation has been shown to improve carbohydrate and lipid metabolism—specifically, insulin, HOMA-IR, triglyceride (TG), HDL cholesterol, and alanine aminotransferase (ALT) concentrations [76].

Whether the association between vitamin D status and MetS is causal remains uncertain. Mendelian randomisation (MR) studies have mostly found no causal effect of 25(OH)D on T2D [77,78] or on MetS and its component traits [79]. MR studies on circulating 25-hydroxyvitamin D and risk of T2D do not consistently support the causal role of vitamin D in T2D in the general population [80], with one exception suggesting modest protection against T2D [81]. In a related context, MR did not support a causal effect on cardiovascular disease or all-cause mortality at any level of 25(OH)D concentrations [82]. Therefore, the observed association may partly reflect confounding and reverse causation. Adiposity may drive low vitamin D status, since bidirectional MR indicates that a higher BMI lowers 25(OH)D, with little evidence of the reverse [83]. To better establish the link between vitamin D status across the various indices of MetS, more high-quality RCTs are required.

### 4.2. Chromium

Essential trace element chromium is required for carbohydrate, lipid and protein metabolism [84], playing a key role in insulin action and glucose regulation [85]. Mechanistically, chromium positively impacts skeletal muscle cell insulin sensitivity and glucose transport regulation via increased activity of the cellular energy sensor 5′ AMP-activated protein kinase (AMPK) [86]. Chromium occurs in the environment primarily in trivalent and hexavalent states, which are beneficial and harmful to humans, respectively [87]. The content of chromium in the human diet is usually low but can be increased through the use of nutraceutical supplements [88].

Supplementation of this nutrient may be important for preventative MetS strategies, given that chromium deficiency is associated with characteristics of MetS, including elevated levels of FBG [89], circulating insulin, cholesterol [90] and TG and a decreased proportion of lean body mass [89,91]. Repleting chromium after experimental dietary depletion led to glycaemic status improvement [91]. Evidence suggests that in those with poorly managed T2D, chromium may improve glucose control in both fasting and postprandial states [92]. In a single-blind randomised clinical trial of those aged 40 to 60 years with uncontrolled diabetes, chromium significantly reduced mean FBG to near-normal levels [93]. Similarly, a case–control study in 4443 Chinese adults examining the associations of plasma chromium levels with T2D and pre-diabetes found an inverse association, meaning lower plasma chromium levels were linked to a higher risk of T2D [94]. Given their favourable safety profile, chromium supplements remain an area of interest in T2D research [95].

During the 23-year follow-up on 3648 American adults from the Coronary Artery Risk Development in Young Adults (CARDIA) study, aged 20–32 years, 878 incident MetS cases were found. Baseline toenail chromium was inversely associated with incidence of MetS, as well as its blood lipid components [96]. Toenail chromium levels were inversely and longitudinally associated with incidence of MetS in American young adults. This inverse association was explained by its relation to blood lipids. In overweight and obese individuals, a meta-analysis of RCTs evaluating the effect of chromium supplementation on cardiovascular-metabolic risk factors found positive effects on weight, insulin and HOMA-IR [97]. Consistent with these observations, a trial of chromium supplementation in coronary heart disease patients observed beneficial effects on weight, BMI, adiposity, and lipid profiles from 12-week supplementation [98]. Further research comparing 2141 MetS cases and 2141 healthy controls during a matched case–control study in Chinese adults found an inverse association between plasma chromium levels and MetS. This was reflected across various MetS indices inversely correlated with plasma chromium levels, including WC, TG, and blood glucose levels [99].

However, findings from clinical trials have been inconsistent. By contrast, no beneficial effect of chromium in a Western population of patients with T2D was reported in an RCT [100]. Similarly, in patients with T2D, neither FBG, weight, nor BMI changed significantly, although HOMA-IR showed a significant decrease (*p* < 0.05), indicating modest beneficial effects on insulin resistance [101]. This demonstrates how variables such as population type, duration, and dosage of supplementation may affect efficacy. Genetic factors or ethnicity may influence the clinical effects of chromium. Therefore, discrepancies in the reported benefits of chromium supplementation should consider subject phenotype based on factors including alterations in chromium metabolism, baseline IR, and the genetics of individuals receiving the supplement [102]. Other factors that complicate the analysis of chromium studies include a lack of reliable analytical tools to assess levels of chromium. For example, blood level only provides insight into a very short-term window of dietary exposure. Sensitivity is further compounded by low levels of unbound chromium circulating in blood (5%), with the majority sequestered in tissue [103]. Alternately, toenail levels provide a historical chromium content that may not well reflect current intake [104]. Together, these represent a major hindrance to understanding the exact role of chromium in these diseases [95]. Although multiple studies report improvements in individual components of MetS, additional clinical investigations must establish appropriate chromium usage and dosage [105].

### 4.3. Probiotics

Habitual consumption of fermented foods has been associated with reduced risks of MetS, CVD, T2D, obesity, and hypertension [106,107,108,109,110,111]. In particular, fermented milk products—namely yogurt—are associated with lower risk of MetS [106,108,111,112,113]. Yogurt is defined as a coagulated milk product resulting from the fermentation of lactic acid by lactic acid bacteria (LAB) *Lactobacillus bulgaris* and *Streptococcus thermophilus* [114]. Many yogurts include additional LAB, such as strains of *Lactobacillus* and *Bifidobacterium*, added for their probiotic effects [115,116]. The benefits of these probiotic LAB are not limited to fermented milk products; human clinical trials of probiotic supplementation in capsule form found similar effects on MetS factors, including decreased body weight, total cholesterol (TC), and plasma TG [117,118].

Current research suggests that 57% of an individual’s gut microbial composition is due to diet [119]. It has been shown that pathological metabolic states, such as obesity and T2D, are associated with dysbiosis of the gut (see Figure 2) [117,120]. Therefore, dietary modification through the use of probiotic supplementation has been hypothesised to be potentially beneficial in the prevention of MetS. A recent review by Green et al. describes a wealth of evidence to suggest ingested strains of *Lactobacillus* and *Bifidobacterium,* alongside prebiotic fibres, may survive the gastrointestinal environment and integrate into the gut ecosystem, leading to metabolic changes such as regulation of the immune system, improved oxidative metabolism, and increased production of SCFAs [117]. Bacterial fermentation within the intestinal lumen produces SCFAs acetate, propionate and butyrate. These play a protective role in health and disease via their actions as G protein-coupled receptor (GPCR) ligands and inhibition of histone deacetylase (HDAC). SCFAs influence, via these signalling pathways, intestinal homeostasis; interstitial energy metabolism (lipid oxidation, gluconeogenesis, glycolysis, and glucose uptake); satiety hormone signalling; and, as most recently identified, inflammation [121].

#### 4.3.1. Probiotics and Gut Function in MetS

In humans, probiotic supplementation has been shown to improve the function of the intestinal barrier and decrease fat mass [122]. A systematic literature review by van Hemert et al. highlighted a potential association between the use of various probiotic strains and decreased intestinal permeability described across 29 human in vivo studies investigating epithelial barrier function. Further, it combined results from a 14-patient case series that explored the effect of a commercially available supplement combining nine different probiotic strains (Ecologic825), including *Bifidobacterium* and *Lactobacillus* species, reporting significant improvements in the integrity of the intestinal barrier [123]. In another study investigating the same probiotic supplement in obese post-menopausal women, improvements were similarly observed in intestinal permeability, as well as fat mass and levels of lipopolysaccharides (LPSs), TC, TG, glucose, and IR [124]. These findings are pertinent, given that post-menopausal women suffer endothelial dysfunction, a shift to central adiposity and lipid changes in the years following the loss of protective oestrogen. Evolving evidence highlights an increased risk of indices like dyslipidaemia and a compromised gut barrier, associated with a reduction in gut microbial diversity. In composite, these increase post-menopausal women’s vulnerability to cardiometabolic disease [125].

Building on these broader findings, a few individual strains have shown promise in supporting gut barrier function. Ballangue and Grill’s study recorded an improvement in intestinal microbial balance, with a notable increase in *Bifidobacterim* abundance alongside reduced levels of putrefying bacteria when human subjects were administered *Bifidobacterium longum* BB536 (BB536) fermented milk [126]. These changes were absent in individuals given fermented milk containing *B. animalis* and all other strains of *B. longum* subsp. *longum* for the same duration [126]. The data suggest the beneficial effects of BB536 on the human intestinal microenvironment are both strain- and host-specific [126]. These findings are further supported by three additional non-RCTs reporting elevated *Bifidobacterium* levels and reduced faecal putrefactive substances [126].

*Bifidobacterium animalis* subsp. *lactis* 420 (B420) has also been shown to improve intestinal barrier function and reduce metabolic endotoxemia, the movement of microbes such as LPS into tissues via the bloodstream from the gut, which leads to reduced inflammation [31,32]. While the mechanism through which microbes translocate from the gut to the bloodstream is unclear, the effects of B420 on intestinal permeability in humans are associated with its ability to increase the levels of beneficial microbes present in the gut and decrease the adherence of bacteria to the intestinal mucus [32].

#### 4.3.2. Probiotics and Metabolic Regulation in MetS

Obesity is characterised by the loss of microbial diversity and increased production of SCFA [127,128]. This allows for more energy to be extracted from the diet, contributing to overnutrition, and is associated with MetS risk factors such as increased adipose tissue, IR, and dyslipidaemia [127,129]. Supplementation with probiotic strains including B420 and *L. acidophilus* has shown regulation of the human gut microbiota and an increase in beneficial bacteria. Thus, probiotic supplementation might reduce the risk of MetS through improved intestinal bacterial diversity [130,131].

*L. acidophilus* 14 (LA-14), in particular, is a promising strain in modulating intestinal microbiota composition. In a liver-injury mouse model, Lv et al. reported that LA-14 increased the abundance of beneficial *Bifidobacterium* species and alleviated d-galactosamine-induced liver injury by preventing the enrichment of *Cyanobacteria* and *Bacteroides*, as well as the depletion of *Oscillospira*, *Coprococcus* and *Lachnospira* [132]. As low levels of 5-hydroxyhexanoic acid are associated with early renal functional decline in T2D, the LA-14-induced increase in this metabolite observed in this study suggests that LA-14 may promote metabolic health [132]. These metabolic benefits of LA-14 are consistent with findings in humans. In an RCT, Gomes et al. found that a probiotic mix, with the addition of LA-14, was more effective in reducing abdominal fat and promoting antioxidant enzyme activity than an isolated dietary intervention in overweight or obese women [133].

In addition, *Bifidobacterium animalis* subsp. *lactis* 420 (B420) has been shown by Stenman et al. to influence body fat mass with and without dietary fibre, and a review by Uusitupa et al. echoed these beneficial effects on obesity, weight management, glycaemia, lipid metabolism, insulin sensitivity and CVD risk [31,32].

#### 4.3.3. Probiotics and Immunomodulatory Function in MetS

Immune dysregulation is one of the main contributors to MetS pathophysiology, and several probiotic strains demonstrate immunomodulatory activity. LA-14 can trigger dendritic cell maturation and activation in vitro, as well as increasing IgG concentrations in vivo, indicating that it influences immune regulation through effects on both innate and adaptive immune responses [134,135]. Similarly, B420 modulates non-specific cellular immune responses, as evidenced by increased phagocytotic activity in an RCT of 26 healthy adult volunteers [136]. More broadly, *L. acidophilus* has demonstrated reduced levels of pro-inflammatory cytokines, improved immunoregulation, and preservation of the intestinal barrier in a number of animal studies [33].

#### 4.3.4. Probiotics and Antioxidant Function in MetS

The role of antioxidants in preventing oxidative stress, as well as the antioxidant insufficiency seen in MetS, is well established [50,137]. Therefore, it has been hypothesised that antioxidant supplementation might be beneficial in the prevention and treatment of MetS [39]. The antioxidant ability of 19 strains of lactic acid bacteria (*Lactobacillus* and *Bifidobacterium*) was tested in vitro by Lin and Yen. All the strains demonstrated scavenging of ROS, metal ion chelation ability, enzyme inhibition, and reducing activity [138]. In relation to reducing oxidative stress in a diabetes mouse model, B420 supplementation was associated with statistically significant reductions in proinflammatory cytokines in mesenteric adipose tissue, as well as normalisation of insulin sensitivity [32,139]. B420 has also demonstrated the ability to reduce inflammation through regulation of the NOS and NFκB pathways in human intestinal epithelial cells [140]. In animal studies, *L. acidophilus* has shown similar positive effects on oxidative stress via modulation of the production of compounds including superoxide dismutase and suppression of the inflammatory response by regulating proinflammatory cytokine levels [33,141,142].

#### 4.3.5. Prebiotics

In contrast to probiotics, which are live microorganisms, prebiotics are “substrates that are selectively utilised by host microorganisms conferring a health benefit” [143,144]. Mainly composed of carbohydrate-based polymers, prebiotics include non-digestible carbohydrates fructo-oligosaccharides (FOS) and galacto-oligosaccharides (GOS); pectic oligosaccharides; starch; and glucose-derived oligosaccharides [143]. In terms of benefit to health, the effects of FOS and GOS as dietary prebiotics have been the most thoroughly documented [144]. Prebiotics first exert their benefits on the host by resisting digestion in the stomach and small intestine, which then allows them to be fermented by the intestinal microflora [143,145]. This fermentation selectively promotes the growth and activity of beneficial intestinal bacteria, such as bifidobacteria, thereby improving overall health [145]. Prebiotics may aid in the prevention of MetS by promoting the production of SCFAs, improving the balance and barrier function of the gut microbiota, and reducing inflammation [143].

#### 4.3.6. Prebiotics and Endocrine Signalling in MetS

Supplementation with dietary prebiotics has been shown to promote bacterial fermentation in the human gut, thereby increasing the production of end products, including SCFAs [145]. SCFAs stimulate the secretion of GLP-1 and peptide YY (PYY) by intestinal L-cells, which act to promote weight loss, satiety, insulin secretion, and b-cell proliferation. In obesity, the plasma concentrations of these hormones is reduced, making further weight gain easier and weight loss harder [146]. Parnell and Reimer found that, when compared to placebo, overweight and obese adults who received a daily supplement of FOS for 12 weeks exhibited decreased body weight, fasting glucose, and insulin concentrations. Participants in the supplement group also self-reported decreased caloric intake, which coincided with increased PYY concentrations and decreased concentrations of ghrelin, a hormone that promotes weight gain and adiposity [147]. Similarly, Dehghan et al. compared women with T2D who were given an inulin supplement for 8 weeks to those given placebo and demonstrated significant decreases in FBG, HbA_1C_, fasting insulin, and IR [148].

#### 4.3.7. Prebiotics and Intestinal Barrier Integrity in MetS

The increase in SCFA production associated with prebiotic supplementation strengthens the intestinal barrier in addition to modulating endocrine signalling [143]. Butyrate improves barrier function by accelerating the assembly of tight junctions between epithelial cells through activation of the AMP-activated protein kinase, as shown by Peng et al. [149]. Gut barrier dysfunction has been connected to microbial dysbiosis [150]. Supplementation with an inulin and FOS mixture for 3 months in obese women led to increased levels of *Bifidobacterium* and *Faecalibacterium prausnitzii*, both of which are associated with decreased serum LPS levels [151]. Furthermore, increased faecal bifidobacteria, in addition to decreased insulin, TC and TG, were observed by Vulevic et al. in overweight adults who received GOS supplementation for 12 weeks [152].

#### 4.3.8. The Role of Prebiotics in Immune System Regulation in MetS

Prebiotics have potential in the prevention of MetS due to their anti-inflammatory properties [143]. SCFAs regulate immunity through activation of G-protein-coupled receptors that reduce the production of inflammatory mediators and inhibit inflammatory pathways. Strengthening of the intestinal barrier prevents LPS entering the bloodstream. LPS triggers NF-κB-mediated inflammatory processes, produces inflammatory cytokines, and has a pro-atherogenic effect [153]. Supplementation with both inulin and GOS has shown anti-inflammatory effects in human and animal studies [143,146,148]. Dehghan et al. reported decreased CRP, TNF-α, and LPS in response to inulin, and Vulevic et al. reported increased secretory IgA and decreased faecal calprotectin and CRP after GOS supplementation [148,152].

#### 4.3.9. Prebiotics in Combination with Probiotics in MetS

In addition to prebiotic supplementation improving metabolic health individually, studies have also reported benefits when taken in combination with a probiotic [154,155,156]. Kassaian et al. gave a daily supplement containing *Lactobacillus acidophilus*, *Bifidobacterium bifidum*, *Bifidobacterium lactis*, *Bifidobacter longum,* and inulin for 24 weeks. In adult prediabetic participants, significant reductions in the prevalence of hyperglycaemia and MetS were reported following treatment [155]. Childs et al. combined *Bifidobacterium animalis* subsp. *lactis* with xylo-oligosaccharide and administered it to healthy adults for 21 days. Increased faecal bifidobacterial counts and decreased inflammatory markers were reported [154]. Finally, Khanna et al. compared *Lactiplantibacillus pentosus* GSSK2 and isomalto-oligosaccharides to orlistat in rats being fed a high-fat diet. After the 12-week intervention, the probiotic and prebiotic combination was shown to have prevented the increase in weight, abdominal circumference, and visceral fat normally seen in high-fat-diet animals. Additionally, increased beneficial bacteria; improved oxidative stress; reduced LPS; and modulation of the inflammatory, lipid, and glucose metabolism genes were reported, highlighting the benefits of the combination in terms of reducing adverse metabolic effects associated with a high-fat diet [156]. Table 1 summarises the available evidence for probiotics, prebiotics and other nutraceuticals, including their proposed functions, nutrient deficiencies reported in individuals with MetS, and reported effects on MetS-related parameters across the populations included in this review.

### 4.4. Antioxidants

Oxidative damage occurs following the metabolism and decomposition of organic fuels within the body. These redox cascades result in the accumulation of ROS that are natural yet associated with the progression of ageing and age-related disease [200]. In MetS, important sources of oxidative stress are mitochondrial, particularly in the heart. In extra-mitochondrial oxidative stress, ROS generation can be upregulated by features under control of the renin-angiotensin system, such as blood glucose, lipids, adiposity, and blood pressure [201]. Therefore, the overproduction of ROS occurs in conditions associated with MetS, such as obesity, IR, hyperglycaemia, chronic inflammation, and dyslipidaemia [202]. Antioxidants are pleiotropically active endogenous molecules produced by cells or are acquired exogenously, protecting cells and tissue from oxidative damage. Though initially understood to primarily impact the redox system via ROS inhibition and scavenging, evolving evidence highlights antioxidant involvement at the cellular transcription level in inflammation, and emerging studies suggest a role for antioxidants in modulating the gut microbiome–immune cell axis. This represents a paradigm shift in the understanding of the scope of antioxidants’ biological effects [203]. Given this, the potential of antioxidant supplementation in support of treatment and prevention of disease indices associated with MetS are widely explored.

#### 4.4.1. General Antioxidant Supplementation in MetS

A large RCT of 5220 generally healthy middle-aged individuals, with data spanning 7.5 years, explored the association between general antioxidant supplementation and risk of developing MetS [204]. While authors reported no association between supplementation and risk in this reportedly well-nourished population, increased MetS risk was associated with low baseline serum concentrations of antioxidants β-carotene and vitamin C. Alternately, baseline zinc concentrations were positively associated with MetS risk. Associations between lower baseline antioxidant concentrations and increased MetS risk likely reflected overall dietary patterns across the population. The results support recommendations for a diet rich in antioxidants [204]. The effect of general antioxidant supplementation on metabolic health indices was explored in a meta-analysis of 27 studies. Authors aimed to clarify if antioxidant supplement consumption might effectively improve metabolic function in obese patients. Outcomes revealed significant decreases in BMI and WC, without effects on the waist-to-hip ratio (WHR), fat mass (FM), or circulating leptin levels in association with general antioxidant supplementation [205]. Compared to placebo, significant reductions in TNF-α were also evident, though not for other inflammatory index markers (IL-6 or CRP). In 17 included studies concerning dyslipidaemia, reductions in TC, TG, and LDL-c levels were observed, alongside increased HDL-c levels. Authors concluded that general antioxidant supplementation modulates metabolic effects in clinical obesity via improved FBG regulation, lower oxidative stress, and reduced inflammation. However, additional research to disentangle which antioxidants are singularly most effective is required [205].

#### 4.4.2. Flavonoids and Polyphenols in MetS

Flavonoids and polyphenols are examples of extra-mitochondrially acting antioxidants with evidence to support their positive effects on indices of metabolic diseases such as hyperinsulinaemia; high cholesterol, blood pressure, and blood glucose; and cardiac hypertrophy [201]. The antioxidant capacity of flavonoids, widely sourced in fruit, vegetables, nuts, and seeds, results from direct scavenging of ROS and an ability to activate antioxidant enzymes like NADPH (nicotinamide adenine dinucleotide phosphate), alongside inhibition of oxidases such as xanthine oxidase (XO) and oxidase regulator PKC. Further, many are understood to act in anti-inflammatory, anti-cardiovascular, anti-bacterial, and anti-fungal capacities [194]. Flavonoid quercetin, found in berries, apples, onions, and red wine, has garnered high-quality evidence relating to its encouraging effects on components of MetS. A 2019 meta-analysis of 16 RTCs reported favourable effects on lipid profiles and inflammatory markers in patients with MetS, significantly reducing TC, LDL-c, and CRP [177]. However, quercetin had no effect on TG, HDL-c, IL-6, or TNF-α levels in these patients. A more recent meta-analysis of 20 RTCs with a total heterogenous sample size of 1164 adults revealed a beneficial effect on FBG and systolic blood pressure (SBP). Similarly, quercetin was not found to benefit lipid markers TG or HDL-c, and it failed to demonstrate a significant effect on WC [178]. Together, evidence suggests quercetin supplementation may have useful effects on some indices of lipid profiles and glycaemic control, but its effects on markers of inflammation and obesity remain equivocal.

Similarly, in a meta-analysis of 10 controlled human trials, resveratrol, a non-flavanol polyphenol compound also sourced in berries, grape skins, and nuts, demonstrated significant improvements in FBG [198]. It was further reported that resveratrol supplementation at doses of >500 mg for >10 weeks yielded further positive effects on WC and TG but not TC levels [198]. A subsequent umbrella review of 11 meta-analyses in patients with T2D assessed wider parameters associated with both MetS and MASLD, including glycated haemoglobin (HbA1c) levels and indices of blood pressure. While results revealed evidence of moderate certainty that resveratrol positively impacted SBP, diastolic blood pressure (DBP), mean arterial pressure (MAP), pulse pressure (PP), HOMA-IR, and FBG, the evidence was weaker for WC and flow-mediated dilatation (FMD) outcomes [196]. Resveratrol was found to have a substantial and clinically meaningful effect on HbA1c levels in patients with T2D, though trials were < 12 weeks. More research is required to assess if the positive effects of resveratrol on glycaemic control are sustained over a longer duration [196]. In patients with obesity and diabetes, a later meta-analysis of 25 RCTs demonstrated a dramatic impact of resveratrol on lipid and glucose metabolism. Here, improvements in WC, HbA1c, LDL-c, TC, and HDL-c levels were reported following resveratrol supplementation, which was proposed to exert positive effects via skeletal muscle, liver lipid, energy metabolism and gut microbiota pathways [197].

Curcumin, a polyphenol component of turmeric (of the ginger family) has some evidence to suggest its supplementation has beneficial effects on indices of glycaemic control and on inflammatory and oxidative stress markers in patients with MetS. In a meta-analysis of 13 RTCs of 785 patients, curcumin exerted significant improvements on WC, FBS, DPB, HDL-c, TNF-α, CRP, and MDA (malondialdehyde) compared to a placebo control [199]. Importantly, the evidence was caveated by a high level of heterogeneity between trials and very low to low–moderate GRADE scores, notwithstanding a reportedly low risk of selection bias. To strengthen conclusions, future studies should encompass diverse ethnicities (10 of 13 studies were conducted in Iran), explore gender differences, and consider the supplement’s formulation in relation to its bioavailability. This is particularly relevant given that curcumin supplementation is often formulated alongside piperine to improve bioavailability, yet its inclusion was noted in only 2 of 13 included RCTs [199].

Polyphenols present in green tea demonstrate emerging promise in MetS prevention. In rodents fed a HFD, the results of supplementation with green tea—specifically, the yellow leaf variety—suggest that polyphenols may modulate the gut microbiota, increase SCFA production, reduce inflammation and obesity, and attenuate MetS [206,207]. Human research is required to clarify these potential benefits.

Artichoke leaf extract (ALE) derived from Artichoke (*Cynara scolymus*) contains flavonoids and caffeoylquinic acid derivatives with reported substantial antioxidant capacity [208]. A recent clinical study revealed that artichoke reduced blood pressure and BMI [209]. In support of this, a meta-analysis suggests artichoke supplementation may offer modest but significant improvements in several cardiometabolic risk markers, supporting artichoke as a complementary strategy for metabolic health. Interestingly, the beneficial effects on blood pressure, liver enzymes, BMI, waist circumference, insulin sensitivity, LDL-c, and TG levels were more pronounced in older, overweight, and obese individuals [210]. Nutraceutical supplementation with polyphenolic fractions of both dry artichoke and bergamot extracts has also been observed to safely exert statistically significant improvements in serum lipids, systemic inflammation, indices of MAFLD, and endothelial reactivity in healthy individuals with suboptimal cholesterol levels [211]. Importantly, ALE supplementation is considered generally safe, with only rare adverse effects reported [211]. Additional long-term clinical trials are required to further verify the protective effects of ALE in MetS and any related complications.

Flavonoids found in the plant extract of bergamot, in combination with other nutraceuticals and alone, are reportedly efficacious in the management of MetS [212]. Bergamot-derived polyphenolic fraction (BPF) significantly reduced serum cholesterol, TG and FBG in patients with dyslipidaemia [213,214]. In patients with MetS and MASLD, BPF also reduced liver steatosis and pro-atherogenic small dense LDL and increased anti-atherogenic HDL-c [215]. While studies have previously focused on the benefits of the purified fraction of bergamot flavonoids [216,217], the entire bergamot phyto-complex comprises a range of bioactive molecules that work in synergy and may exert a stronger effect than the single active fraction, presenting a potential avenue of clinical interest [218]. A recent double-blind three-arm RCT reported significant improvements in glucose and lipid metabolism, as well as inflammation, after 12 weeks of bergamot phyto-complex in individuals with MetS [195]. These findings highlight natural extracts as a promising multi-target solution for enhancing cardiometabolic health. Moreover, ALE and bergamot are generally safe, with only 2% of individuals experiencing mild and transient AEs, including dyspepsia, following supplementation [211,219]. To better understand the protective effects of ALE and bergamot in MetS and its related conditions, additional long-term clinical trials are required.

#### 4.4.3. Carotenoids and Vitamins in MetS

Carotenoids, which are ubiquitous in human serum, are antioxidants found in fruit, vegetables, eggs, fats, and oils and are precursors to vitamin A (retinol). Retinol plays a role in transcriptional regulation of cell-cycle progression to support cell and tissue maintenance. In the general population, evidence identified an inverse association between MetS and serum levels of α- and β-carotene, as well as retinyl esters [220]. Evidence from a Korean population-based RTC of 6308 adults indicates that increased dietary intake of vitamin A and vitamin E was inversely associated with risk of MetS, demonstrating improvements in central obesity and HDL-c levels [221]. In the prevention of cardiometabolic disease indices, these results highlight a necessity for formal recommendations regarding daily intake of vitamin A and its precursors. Owing to their antioxidative properties, other vitamins commonly studied include vitamins B, C, D and E. In a meta-analysis of 12 RTCs assessing oxidative stress and glycaemic control in patients with T2D, vitamin E supplementation was found to be related to significant reductions in blood glucose and glycated haemoglobin, while the antioxidant properties of vitamins E and C concomitantly increased superoxide dismutase enzyme (SOD) and total antioxidant capacity (TAC) status compared to placebo controls [222].

Lycopene is an exclusively carotenoid sourced exclusively from the diet and highly concentrated in tomatoes. Rather than being converted into vitamin A and its derivatives, lycopene undergoes oxidation and enzymatic metabolism into apo-lycopenals and apo-lycopenones. A recent meta-analysis presented pooled evidence of 29 RCTs identifying a significant inverse association between serum lycopene levels and risk for MASLD, suggestive of a role for lycopene in liver protection and indicative of low dietary intake in obese individuals [181]. However, owing to substantial heterogeneity between trials, evidence of lycopene’s effects on other MetS indices such as glycaemic control and obesity were less certain [181]. In a recent umbrella review of five meta-analyses assessing the role of lycopene sourced from the diet in cardiovascular risk, the authors reported high-certainty GRADE evidence of significant yet clinically modest improvements in blood pressure following tomato-derived lycopene consumption (2–3 mmHg reduction in SBP [182]). Notably, these effects followed intake of 5–30 mg of lycopene, equating to the daily consumption of just one or two tomatoes. Reasonably, consuming tomatoes will also increase exposure to an abundance of additional antioxidants, including quercetin, that could exert synergistic effects to improve vascular function. Mechanistically, lycopene’s effects on blood pressure well reflect our understanding of its antioxidant actions in enhancing vascular function via endothelial NO production and the inhibition of angiotensin-converting enzyme (ACE) activity. In this umbrella review, heterogeneity between meta-analyse’ resulted in pooled evidence on lipid profile markers that were less discernible [182]. Taken together, evidence may suggest lycopene is liver-protective, with an additional—albeit clinically modest—impact on blood pressure. Given that lycopene is easily sourced, its addition to the diet is feasible for many individuals, requiring only minimal nutritional adjustments.

#### 4.4.4. Coenzyme Q10 in MetS

Antioxidant Coenzyme Q10 (CoQ10), also known as ubiquinone, inhibits lipid peroxidation within mitochondria and is consequently present in most aerobic organisms. Sourced in small amounts in oily fish, meat, nuts, oils, and vegetables, CoQ10’s primary role relates to its ability to facilitate electron transfer to regulate energy metabolism and the electrochemical membrane gradient [172]. Owing to its role in intracellular oxidation, there is vast interest in CoQ10 supplementation and markers of oxidative stress and inflammation, particularly in relation to indices of MetS. A 2020 meta-analysis of 17 RTCs assessed parameters of oxidative stress in response to supplementation with CoQ10 and reported significant reductions in MDA concentrations alongside increased TAC and antioxidant defence-system enzymes [223]. Notably, the increase in TAC was significantly more prominent within the diabetic sub-group. The effect was also more evident in senior adults, in doses >200 mg, and in studies lasting >8 weeks. There was no effect of CoQ10 supplementation on NO production, antioxidant glutathione (GSH), or catalase (CAT) activity [223].

Evidence of the effects of CoQ10 supplementation on lipid profile in patients with MetS also demonstrates promise. A meta-analysis of 318 patients with noted high-quality evidence (GRADE) reported significantly increased adiponectin levels alongside reductions in inflammatory markers IL-6, CRP, and TNF-α in those supplementing with CoQ10 [176]. Mechanistically, metabolic regulator adiponectin impacts pro-inflammatory markers associated with low-grade inflammation in obesity by reducing leptin, improving glucose regulation and enhancing lipid metabolism [173]. In combination with Monacolin-K, CoQ10 supplementation supported an improvement in lipid profile, specifically lowering LCL-c and TG levels alongside blood pressure in an RCT of individuals with MetS [224]. However, in later studies exploring red yeast rice extract in Japanese populations, low-dose Monacolin-K demonstrated promise in treating indices of dyslipidaemia and blood pressure without CoQ10 [225]. It has since been proposed as a possible alternative to statin therapy. However, progress has been hampered by its likely contamination with mycotoxin Citrinin, requiring rigorous quality control by supplement manufacturers [226,227,228].

A subsequent umbrella analysis of 13 meta-analyses did not assess the effects of CoQ10 on adiponectin levels but concurred with prior evidence that supplementation significantly improves various markers of oxidative stress—specifically, CRP, MDA, TAC, and SOD [175]. Moreover, a weighted analysis of subgroups revealed significant improvements in inflammatory markers TNF- α and IL-6, as well as CRP levels. Here, the effects of CoQ10 supplementation were more pronounced in younger adults (<55 y) and in studies longer than 10 weeks [175]. Together, evidence suggests that CoQ10 may be an effective complimentary therapy in the treatment of chronic inflammation and dyslipidaemia, conditions understood to contribute to the progression of MetS disease indices. Evidence indicates that the dosage of CoQ10 and duration of supplementation, alongside age and diabetic status, may be of consequence with respect to its efficacy.

#### 4.4.5. Choline

Choline, now recognised as an essential nutrient, is integral to the regulation of phospholipid synthesis, including serum and membrane lipid composition, and is involved in membrane transport, muscle structure and function, and fat and protein metabolism [162,163]. In 2019, choline was identified as an agonist in GPCR activation (SigmaR1), revealing its potential as a therapeutic target in Ca^2+^ modulation [229]. It serves as a precursor to neurotransmitter acetylcholine, betaine, phosphatidylcholine and trimethylamine (TMA). Based on its form, dietary choline can be lipid- or water-soluble, affecting its bioavailability and metabolism.

Given that key dietary sources include animal-based produce like eggs, fish, beef, and milk, deficiencies in choline can stem from a vegetarian or vegan diet, though choline is also rich in beans and cruciferous vegetables [162]. Deficiencies are also common in pregnancy and lactation, owing to an increased demand for choline unmatched by dietary intake. Maternal choline deficiencies are associated with foetal neurodevelopment issues, specifically in brain function and memory, but also in the liver and metabolism [162,166]. In adulthood, choline-deficient individuals are at greater risk for MASLD [164]. Expectedly, choline is a common addition to food supplements.

Evidence regarding choline’s role in the development of, or protection from indices of metabolic syndrome are conflicted. Compelling evidence exists to suggest moderate dietary intake reduces CVD risk concurrent to a reduction in all-cause mortality [230]. However, evolving knowledge also reveals a complex interplay between increased choline, the gut microbiota and an increased risk of atherosclerosis [163,164]. In line with this conflicting landscape, a recent meta-analysis of five studies identified a positive linear association between diabetes incidence and dietary choline or betaine intake. Notably, the association was non-significant, and the review included trials with substantial risk of bias, high heterogeneity, and very low-quality GRADE evidence [231]. Nevertheless, the difference in risk between choline deficiency and choline excess remains unclear and warrants further investigation.

The American Heart Association presented evidence that choline is beneficial in the PREDIMED (Prevention with Mediterranean Diet) study, demonstrating an inverse association between serum choline metabolites and risk for cardiovascular disease (2.27-fold higher risk in those with the lowest choline metabolite score). For individuals in the lowest choline metabolite tertile, increased CVD risk was attenuated by an intervening Mediterranean diet, suggesting that a healthy diet can mitigate some of the consequences associated with choline deficiency [165]. In another study exploring natural dietary choline, a small, randomised cross-over trial assessed the effect of egg intake (3 per day) versus a choline supplement on indices of metabolic health in individuals with MetS (n = 23) [168]. After 4 weeks, there were no changes to lipid profiles for either treatment compared to baseline, but inflammatory marker IL-6 was reduced following both. Additionally, the egg group demonstrated lowered CRP, insulin and IR compared to baseline, suggestive of additive effects of other micronutrients present in eggs, highlighting them a good dietary choice rich in choline for individuals with MetS [168].

The association between dietary choline or betaine intake and cardiometabolic risk in obese individuals was explored by Abbasi et al. Those in the highest tertile exhibited increased indices of obesity (BMI and WHR). Conflictingly, increased fat free mass (FFM) and basal metabolic rate (BMR) were evident in the same subset [169]. Given the recognised inaccuracies of BMI as a measure of health, these results could be reflective of choline-associated positive changes in body composition rather than adiposity [232]. Moreover, for individuals with the highest intakes of both choline and betaine, blood pressure and LDL-c significantly improved. Together, this evidence may suggest that dietary choline and betaine are additive in protection against indices associated with MetS risk [169].

In recent years, our understanding of the potential risks associated with excess choline on cardiometabolic indicators has evolved further. Central to this is the conversion of choline to its metabolite, TMA, facilitated by gut microbes in the small intestine. In the liver, TMA is oxidised to TMAO. Though TMAO can be excreted, in excess it accumulates in the circulation and tissues where it may contribute to adverse effects on cardiometabolic factors associated with metabolic syndrome [164,170]. TMAO levels are understood to be an independent risk factor for cardiovascular events across multi-ethnic cohorts, owing to its direct involvement in atherosclerotic plaque development [171]. In addition to an increased risk of stroke and myocardial infarction (MI), increased levels of TMAO are also implicated in endothelial dysfunction and impaired renal function [164,170,171].

Microflorae are an attractive target within this metabolic chain, and efforts to influence the diet–microbiome axis are underway. Consequentially, studies have explored the effect of various therapies on TMAO production including resveratrol, SCFAs, probiotics, allicins and antibiotic therapy, with varied success (reviewed by Arias et al.) [170]. Notably, a diet rich in prebiotics via soluble fibre demonstrated reduced TMAO metabolism by 62% in mice fed a diet rich in red meat [233]. A recent meta-analysis reported consistent changes in TMAO production following exposure to genera such as *Akkermansia* and *Bifidobacterium*, as well as prebiotic interventions that concomitantly support the diversification of the gut microbiota [234]. Studies comparing sources of dietary choline have measured differential effects on gut biodiversity and their potential impact on TMAO levels. However, no difference in microbiota diversity or TMAO production was reported between egg-based choline, supplemental choline, and baseline. However, increases in serum carotenoids lutein and zeaxanthin following egg consumption indicate enhanced antioxidative properties [235].

Taken together, evidence suggests that neither deficiency nor excess of choline is desirable in prevention of metabolic syndrome. Striking a balance of choline intake via a varied diet rich in fibre appears to be key. Moreover, diversifying supportive gut flora in composite may well reduce the metabolism of TMAO and associated CVD risk.

#### 4.4.6. Isoflavones in MetS

Soy is rich in phytoestrogenic isoflavones, such as genistein, which exert oestrogen-like activity through oestrogen receptors [236,237,238,239]. Oestrogen influences both insulin sensitivity and lipid metabolism. Given this, research suggests that isoflavones may help to improve metabolic outcomes via oestrogenic activity, particularly in postmenopausal women [240,241,242].

Subsequent research has since examined this population more robustly. A cross-sectional analysis of 5107 adult women reported that soy consumption is associated with lower risk of increased WC, elevated TGs, reduced HDL-c, and increasing BP [238]. This was supported by a double-blind RCT in 60 postmenopausal women with T2D, supplementing 54 mg genistein daily for 12 weeks [236]. In this short timescale, genistein supplementation significantly reduced FBG, HbA1c, TG, MDA and TAC while significantly improving HDL-c and IR [236].

Longer-term evidence from a placebo-controlled RCT spanning 12 months and involving 120 post-menopausal women with MetS supports these findings [237]. Those supplementing daily with genistein also demonstrated significant reductions in FBG, fasting insulin, and HOMA-IR. Importantly, these improvements were not reflected in placebo controls [237]. Moreover, women consuming supplemental genistein demonstrated significant improvements in HDL-c and adiponectin levels, while TC, LDL-c, TG, visfatin, and homocysteine levels, as well as systolic and diastolic blood pressure, were concurrently reduced [237].

#### 4.4.7. B Vitamins and MetS

Water-soluble B vitamins, including vitamins B_3_ (niacin), B_9_ (folate) and B_12_ (cobalamin), play essential roles in cellular metabolic pathways, including DNA methylation and regulation of one-carbon metabolism for the homeostasis of both amino acids and lipids [157]. As such, their deficiency can lead to significant health consequences, including cellular inflammation and elevated synthesis of fat and amino acid homocysteine [158]. Abnormally high levels of homocysteine (hyper-homocysteinaemia) can contribute to the development of IR [179], as well as cardiovascular and cerebrovascular diseases via atherosclerotic vascular–endothelial injury [243]. Importantly, some studies have shown that folic acid and vitamin B_12_ supplements are effective in reducing plasma homocysteine levels [244]. Given this, B-vitamins may help to reduce the prevalence of a variety of health indices associated with MetS. Further, impaired glucose oxidation due to mitochondrial dysfunction plays a crucial role in MetS. Since B vitamins are crucial for optimal mitochondrial function and overall metabolic processes, particularly within the context of MetS, they may offer preventative properties. While the rationale for vitamin B to support metabolic health is mechanistically plausible, the supportive data are inconsistent where population variability remains a crucial factor.

#### 4.4.8. Niacin and MetS

Niacin plays a crucial role in cellular metabolism [245]; improves endothelial function [246]; and is beneficial in lowering blood lipids [247], including atherogenic lipoproteins containing apolipoprotein-B [248]. It has been used for more than half a century to treat dyslipidaemia [249]. However, in recent years, its use in CVD prevention has diminished, owing to reports of variable efficacy and an unfavourable side-effect profile [250]. Niacin may be useful in the treatment of MASLD and its consequences [249]. Taken together, its protective effect in MetS remains unclear.

An analysis of the National Health and Nutrition Examination Survey (NHANES) (2007–2014) examined the relationship between B vitamins, including B_1_, B_2_, niacin, B_6_, B_12_ and dietary folate equivalent (DFE), and MetS. Moderate intakes of vitamin B_1_ and B_12_ and higher intakes of niacin and vitamin B_2_ were associated with lower TG. Niacin, as well as higher intakes of vitamin B_1_, B_2_ and B_6_, may increase levels of atheroprotective HDL-c [248,251]. Niacin was also inversely associated with elevated FBG and abdominal obesity, the latter of which was also observed in association with higher intakes of vitamins B_1_, B_2_, B_6_, and DFE. The inverse relationship found between dietary vitamin B_1_, niacin, B_6_, and DFE and MetS was described as linear, whereas the relationship between dietary vitamin B_2_ and MetS was termed non-linear inverse. The results discussed here indicate that higher intake of vitamins B_1_, B_2_, and B_6_; niacin; and DFE may be associated with reduced risk of MetS [251], suggesting a possible role in the prevention of MetS.

Further analysis of the NHANES database, representative of the US population, investigated the long-term effects of niacin intake on all-cause and cardiovascular mortality rates among individuals with MetS. Results revealed higher dietary niacin intake is associated with reduced cardiovascular and all-cause mortality risks in this population, in an apparent dose–response relationship [252]. Regardless of MetS status, niacin monotherapy decreased the occurrence of 6-year MI and 15-year total mortality in men with previous MI with or without MetS [253].

However, contradictory results reveal adverse effects of niacin on the cardiovascular system, caused by breakdown products of excess niacin, 2PY and 4PY, which promote vascular inflammation [254]. One study reported increased risk of hypertension in those with a daily niacin intake exceeding 15.6 mg/d [255]. This is in line with multiple meta-analyses that question whether niacin prevents CVD [256,257]. Since niacin has complex effects on lipids and CVD, additional large-sample studies among individuals at risk of developing MetS are required to clarify its role.

#### 4.4.9. Vitamin B_12_ and Folic Acid in MetS

Several large studies reinforce a protective role for folate and vitamin B_12_ with respect to MetS and its components. A prospective study over 30 years observed an inverse association between MetS and intakes and serum levels of folate, vitamin B_6_, and vitamin B_12_ among Black and White young adults in the US (n = 4414). Participants with sufficient folate intake were 61% less likely to develop MetS [159]. Similarly, a cross-sectional analysis of 6633 participants with overweight/obesity and MetS suggests that higher folate intake may be associated with a lower MetS score in older adults, owing to a lower reported FBG and a greater level of HDL-c in high-risk cardio-metabolic subjects [258]. Mechanistically, folate and vitamin B_12_ may have beneficial effects on CVD risk factors, owing to their actions on IR and endothelial dysfunction, alongside their ability to decrease homocysteine levels in patients with MetS [259].

In patients with T2D, vitamin B_12_ supplementation as an add-on to stable oral glucose-lowering therapy has been shown to improve glycaemic control and IR, both MetS-related traits [161]. In this context, long-term metformin use in the Diabetes Prevention Program (DPP)/DPP Outcomes Study (DPPOS) has been associated with biochemical B_12_ deficiency and anaemia, highlighting a possible need for routine testing of vitamin B_12_ levels in metformin-treated patients and vitamin B_12_ supplementation [260]. In hypertensive patients, a meta-analysis of RCTs suggests that high-dose folic acid supplementation reduces blood pressure (BP), a core component of MetS [160]. In patients with MetS, findings from a meta-analysis on RCTs suggest folic acid supplementation could reduce inflammatory markers associated with MetS, including TNF-α, IL-6 and hs-CRP [167].

On the contrary, a meta-analysis investigating the efficacy of folic acid supplementation in the prevention of CVD found that although supplementation significantly reduced stroke risk, it had no significant effect on coronary heart disease or peripheral arterial disease. Here, folic acid supplementation did not affect lipid profiles and had no significant impact on HDL or LDL levels [180]. Among women at high-risk for CVD, a combination of folic acid/vitamin B_6_/vitamin B_12_ did not reduce a combined endpoint of total cardiovascular events, despite demonstrating significantly lower homocysteine [261]. Similarly, a post hoc analysis of the China Stroke Primary Prevention Trial (CSPPT) found no significant effect of folic acid supplementation on the risk of new-onset diabetes in 20,702 hypertensive adults without history of stroke or MI [262].

Taken together, evidence of a role for B vitamins in the development and prevention of the indices associated with MetS remains conflicting. Future research across populations with increased diversity may help to better elucidate their role.

## 5. Toxicity and Safety of Nutraceuticals

The growing popularity of nutraceuticals in the prevention and management of metabolic disorders raises important safety concerns that may limit their clinical application. A primary issue is dose-dependent toxicity, whereby excessive consumption of bioactive compounds can result in adverse health outcomes. Many nutraceuticals are perceived as inherently safe because they may derive from natural sources. However, high-dose supplementation has been associated with risks including hepatotoxicity; increased bleeding tendencies; and, in rare cases, fatalities [263,264]. For example, a systematic review and meta-analysis reported that omega-3 polyunsaturated fatty acid supplementation, particularly at higher doses, may be associated with a small but measurable increase in bleeding risk [265].

Drug–nutraceutical interactions represent another important safety consideration. This is particularly relevant to MetS patients prescribed multiple medications (polypharmacy). Some nutraceutical compounds could influence drug metabolism through effects on cytochrome P450 (CYP450) enzymes and membrane transporters, potentially altering drug bioavailability and therapeutic activity. Such interactions may increase drug toxicity or reduce the efficacy of prescribed medications, creating significant challenges for clinical management [263]. Moreover, CYP450 enzyme induction can accelerate drug metabolism and diminish therapeutic efficacy. These interactions are of particular concern in patients exposed to polypharmacy.

An example of a nutraceutical with known consequence on liver metabolic enzymes can be derived from grapefruit juice, a well-established modulator of drug metabolism due to its potent inhibition of intestinal cytochrome P450 3A4 (CYP3A4). This enzyme is responsible for the first-pass metabolism of numerous medications, including statins, calcium-channel blockers, and several immunosuppressive agents [266]. Inhibition can increase the systemic bioavailability and plasma concentrations of affected drugs, thereby enhancing risk of adverse effects. In the case of simvastatin, concurrent consumption of grapefruit juice has been shown to substantially elevate plasma drug concentrations, increasing the likelihood of statin-associated myopathy and, in severe cases, rhabdomyolysis [267]. Consequently, grapefruit extract supplements may have the potential to produce drug interactions similar to those observed with grapefruit juice, increasing the systemic exposure of medications metabolised by CYP3A4, including statins, calcium-channel blockers, and immunosuppressants. However, the magnitude of such interactions may vary depending on the composition, standardisation, and concentration of bioactive constituents within the supplement formulation [268].

Experimental evidence suggests that curcumin and several flavonoids can also inhibit cytochrome P450 enzymes, particularly CYP3A4 and CYP2C9, which may interfere with the metabolism of concomitantly administered medications and increase the potential for clinically relevant drug–nutraceutical interactions [269,270]. Some compounds induce CYP450 enzyme production, increasing metabolic activity to facilitate rapid drug clearance and reducing drug effectiveness. St John’s Wort is an example, inducing CYP3A4 to accelerate metabolism of numerous compounds. Marketed ‘herbal products’ and ‘botanical extracts’ have also been shown to influence CYP1A2 and CYP2C19 activity [271].

The concurrent use of several medications increases the risk of clinically significant drug–nutraceutical interactions, particularly those mediated through cytochrome P450 (CYP450) enzymes and membrane transporters involved in drug metabolism and disposition [36]. Commonly used nutraceuticals for metabolic health, including berberine, curcumin, green tea extract, resveratrol, and omega-3 fatty acids, have been reported to modulate CYP450 activity and may therefore alter the pharmacokinetics of co-administered medications [36,272].

Evidence regarding the excessive use of curcumin and its potential adverse effects on multiple organ systems raises concerns. For example, Liu et al. reported potential toxicological concerns associated with curcumin overuse, including effects on hepatic, cardiovascular, renal, and immune function [273]. In the context of MetS, both clinicians and patients should remain vigilant regarding possible drug–nutrient interactions and the risk of physiological stress or toxicity associated with curcumin supplementation.

Additional concerns have been raised regarding the potential association between curcumin supplementation and nephrolithiasis. Baxmann et al. suggested that supplemental curcumin may contribute to an increased risk of kidney stone formation, while others have reported elevated urinary oxalate levels following curcumin consumption, particularly among susceptible individuals [274,275]. These findings indicate that certain populations, including those with MetS, who may already present with metabolic and renal vulnerabilities, could be at increased risk of adverse effects associated with curcumin supplementation.

Furthermore, studies have highlighted potential effects of curcumin on iron metabolism. In murine models, Jiao et al. reported that curcumin induced iron deficiency, raising questions regarding its impact on iron homeostasis [276]. Similarly, other rodent studies emphasised the need for further investigation into the implications of curcumin use among individuals with existing iron deficiency or impaired iron status [277].

Drug–nutrient interactions represent another concern. Examining the pharmacokinetics of tamoxifen, co-administration with curcumin was reported to alter drug exposure, potentially affecting therapeutic efficacy [278]. This finding highlights the clinical significance of interactions between nutraceuticals and prescribed medications. Given that individuals suffering from MetS—or related indices—may also be undertaking treatment for comorbidities, the potential for such interactions should not be overlooked. A holistic approach to patient care is therefore essential, whereby the benefits and risks of curcumin supplementation are evaluated within the broader context of an individual’s overall health status, comorbidities, and concurrent pharmacological treatments.

Furthermore, increasing reports of nutraceutical supplement-induced liver injury have raised concerns regarding the potential adverse effects of nutraceutical products. In the United States, nutraceutical supplements are now recognised as a growing cause of drug-induced liver injury, accounting for an increasing proportion of hepatotoxicity cases [279]. More recently, a case report of drug-induced liver injury associated with a turmeric supplement containing piperine further highlighted the potential hepatotoxic risks of concentrated turmeric-derived nutraceuticals [280]. These findings suggest that adverse events associated with nutraceutical use may be more prevalent than previously recognised and emphasise the importance of enhanced post-market surveillance, adverse event reporting systems, and regulatory monitoring [279].

Additional concerns arise from the limited regulatory oversight and lack of standardisation within the nutraceutical sector. Internationally, nutraceutical products are marketed without undergoing pre-market evaluation equal to that required for pharmaceutical agents [263]. Consequently, issues such as contamination, mislabelling, inaccurate dosage declarations, and variability in active ingredient concentrations have been widely reported, posing potential risks to consumers [263]. Evidence regarding the long-term safety of nutraceutical use remains limited, particularly in chronic conditions where supplementation may continue for months or years, alongside polypharmacy. Variability in individual responses, influenced by genetic characteristics, metabolic status, and gut microbiome composition, further complicates the prediction of safety outcomes and therapeutic effectiveness [263].

Within the European Union (EU), nutraceuticals are generally regulated as food supplements under Directive 2002/46/EC, with product safety and health claims evaluated through scientific risk assessments conducted by the European Food Safety Authority (EFSA). This framework adopts a precautionary approach, requiring authorisation for novel ingredients and substantiation of health claims before market approval. In contrast, the United States regulates nutraceuticals under the Dietary Supplement Health and Education Act (DSHEA) of 1994. Under this legislation, manufacturers are primarily responsible for ensuring product safety, while regulatory authorities rely largely on post-market surveillance rather than mandatory pre-market approval. As a result, products may reach consumers without the extensive safety and efficacy evaluations typically required for pharmaceutical agents. Regulatory frameworks across the EU, the United States, and the United Kingdom are compared in Table 2.

The absence of internationally harmonised definitions, quality standards, and regulatory requirements remains a significant challenge, contributing to variability in product composition, safety assurance, and clinical integration. Consequently, greater international collaboration and regulatory standardisation are needed to improve consumer protection and support the evidence-based use of nutraceuticals in clinical practice [263,281]. Santini et al. (2018) argued that the absence of a universally accepted definition and regulatory framework for nutraceuticals presents significant challenges for ensuring product quality, safety, efficacy, and clinical integration [282].

## 6. Discussion

Not long ago, abdominal obesity, IR, dyslipidaemia, and hypertension were viewed as independent entities. Our understanding that their presence in composite represented a syndrome evolved following the frequent identification of cardiometabolic symptom clustering in affected individuals. Now, their coexistence and additive effects on morbidity and mortality are better understood. Consequently, there is a pressing global need to prevent this complex collection of diseases and to reduce its associated socioeconomic burden. Supervised nutritional optimisation and long-term maintenance of a low BMI can reduce the prevalence of MetS. Medical and surgical treatments such as the use of GLP-1 agonists and bariatric surgery have a role where conventional measures have failed. They are, however, not without side effects and require regular monitoring. Both GLP-1 receptor agonists and bariatric surgery can produce sustained appetite suppression and delayed gastric emptying, raising concerns about their long-term impact on micronutrient and vitamin status [283]. This underscores the need for enhanced nutritional education and shared responsibility between prescribers and patients. Surging GLP-1 agonist use is also causing a disproportionate strain on primary care drug budgets in some countries, and weight regain is common after stopping medication. A low-cost, safe, and widely applicable approach is needed. Nutraceuticals, both dietary and ingested as a supplement, together with lifestyle changes, may be part of the answer. The vast interest in this topic on the part of industry, academia and governmental organisations is instructive. There may be a trend emerging to offer specific evidence-based nutraceutical combinations that positively influence a particular pathological process rather than recommend general multivitamin/mineral supplementation.

There are many examples of nutraceuticals demonstrating beneficial effect (Table 1.). This review highlights current research on CoQ10 supplementation, owing to its role in oxidative stress and inflammation, particularly in relation to indices of MetS [284]. Significant improvements in blood pressure were demonstrated following tomato-derived lycopene consumption [182].

The majority of an individual’s gut microbial composition is due to diet and environment. Because pathological metabolic states, such as obesity and T2D, are associated with dysbiosis of the gut, dietary modification using prebiotic and probiotic supplementation, alongside fibre-rich and fermented produce, have the potential to be beneficial in the prevention of MetS. Evidence suggests these are associated with increased SCFA production and microbial diversity, as well as reduced LPS production and inflammation, resulting in decreased body weight, TC, and TG and increased insulin sensitivity [55,111]. Notwithstanding the effects of ultra-processed food on gut diversity discussed here, it may also be instructive to reflect on increasing antibiotic usage and the resultant, often prolonged gut dysbiosis [56].

While this review aimed to assess the role of nutraceuticals in MetS prevention, studies in this area were lacking in comparison to those investigating the role of nutraceuticals in managing established indices of MetS such as T2D, obesity and CVD. Given the multicomponent nature of this condition, it was pertinent to explore the wider literature, across all contexts, while noting where quality evidence supports preventative measures across deficient populations. Importantly, this highlights the need for future research focussed on the role of nutraceuticals in the prevention of metabolic syndrome, specifically in those with established nutrient deficiency. Current research on nutraceuticals for MetS is expanding rapidly. Because gut dysbiosis with associated alterations in permeability, microbial pathways and chronic inflammation are central features of MetS, these, in turn, may offer additional therapeutic opportunities. However, important knowledge gaps remain. These problems span mechanistic understanding, clinical evidence, and how to translate their use into clinical practice. Moreover, we do not fully understand all the pathophysiological processes involved in the development of MetS. Chronic inflammation, oxidative stress, adipokine dysregulation and gut health play a role [35].

Similarly, we do not know how many nutraceuticals exert their effects, particularly regarding interactions with metabolic pathways, other treatments and individual variability driven by genetic and microbiome differences. The role of the gut and processes that modify inflammation are likely to be important [285]. One important and consistent finding of this review is that nutrient deficiencies are often associated with the development of various indices associated with MetS. These associations have been highlighted in choline [165], B vitamins [159], chromium [89], and vitamin D [66], indicating an important role for evidence-based intervention. Given this, it is pertinent to highlight the importance of re-testing deficiencies following alterations to diet and supplementation. In careful and tailored exposure, increased risk of adverse effects as a consequence of excess supplementation may be avoided.

Another problem is that research in this area is often of poor quality. Many nutraceuticals show promising preclinical or small-scale clinical results, but robust, large RCTs with long-term follow-up are scarce [286]. Existing studies often focus on surrogate outcomes (e.g., lipid levels or glucose markers) rather than hard clinical endpoints such as cardiovascular events or mortality [286]. Evidence is frequently heterogeneous or inconsistent, particularly in obesity and diabetes research [286]. This is not surprising in view of the challenges of controlling variables, defining appropriate endpoints, blinding, and upholding ethical practice. Further issues when comparing study data include nutraceutical composition, dosing, and bioavailability, all of which complicate interpretation and reproducibility. These considerations partly account for nutraceuticals only being minimally integrated into clinical guidelines [286]. Collectively, these limitations highlight the need for more rigorous, standardised, and translational research to clarify their therapeutic role [287].

Nutraceuticals, as a therapeutic option, are at risk of being dismissed or marginalised because of a lack of scientific research, as well as worries of toxicity and safety, but also because of concerns over product quality control. Composition and consistency can alter efficacy and toxicity risk. Similarly, added ingredients may cause intolerance and interfere with absorption [279]. Understandably, wherever there is a lack of knowledge, there is misinformation. This opens the door for unsubstantiated marketing claims and endorsements via social media platforms.

## 7. Conclusions

This review suggests that some nutraceuticals, obtained from dietary sources or as supplements, may have a role in prevention and management of metabolic syndrome alongside other lifestyle measures. Crucially, reported improvements across metabolic parameters should be interpreted cautiously, and further high-quality research is needed to establish their clinical relevance and to clarify the role of supplementation in those with nutrient deficiencies and chronic disease. They may eventually prove to be a viable preventive option in regions without ready access to therapies available in affluent economies and for vulnerable or high-risk populations in whom access to specialist care, pharmacological therapies, or intensive lifestyle interventions is limited. Unfortunately, to date, there are limited guidelines as to their usage, though the evidence base is evolving, particularly in regard to the role of gut microflora diversity and maintenance of intestinal barrier integrity. Many individuals take regular supplements with the aim of improving health without clear evidence as to benefit. Key questions persist regarding the optimum agent alone or in combination, duration of use, timing of administration, potential synergistic effects with other interventions such as exercise or co-administered agents, and the identification of populations most likely to benefit. Like medicine, these are not innocuous, can infrequently cause side effects and may interact with other agents. Therefore, it is important to obtain nutraceuticals from a reputable source; take them as advised; not exceed the recommended dose; and consult a dietician or HCP if you are pregnant, breastfeeding, managing chronic conditions such as chronic kidney disease or diabetes, or taking prescribed medications. They are intended to complement, not replace, evidence-based lifestyle modifications. They cannot currently be recommended as the sole agent to prevent or treat MetS.

Further research is warranted in the face of this pandemic, with a study design that answers detailed clinical questions specifically concerning endpoint data, formulations, and dosing. Such studies are challenging and expensive to conduct but especially necessary as GLP-1 agonist usage increases, becoming a more accessible and viable clinical choice for people at risk of MetS and associated conditions.

## Figures and Tables

**Table 1:** A summary of evidence relating to nutraceuticals and metabolic syndrome.

Supplement	Proposed Function	Food Sources	Deficiency in MetS	Reported Primary Outcomes	Evidence Strengths and Limitations
**Vitamin B** **_12_**	DNA synthesis and cellular energy production [157].	Meat, fish, dairy, eggs, poultry, yeast extract, fortified foods like cereals and plant milk	Deficiency associated with MetS and long-term metformin use [158].	**In general adults:** Preventative ↓ MetS incidence [159] **In high-risk CVD (women):** ↓ Homocysteine ≠CVD events [160] **In T2D:** ↑ Glycaemic control, IR [161]	Long-term, large, prospective, multi-racial study evidence; meta-analysis in patients with MetS
**Vitamin D**	Supports glycaemic control; associated with immunomodulation, visceral fat deposition, and dyslipidaemia [73]	Oily fish, red meat, eggs, fortified foods like cereals and plant milk	Deficiency is common and associated with adverse metabolic parameters across multi-country and multi-ethnicity studies. ↑ risk for MetS, NB: association unlikely to be causal [77,79]	**In older adults:** ≠WC, weight, body mass index (BMI), total fat mass and lean tissue (individuals aged 50 and above) [67] **In Vitamin D deficiency:** Improved IL-6, HOMA-IR, BMI, WC (postmenopausal women, PCOS women with obesity or insulin resistance) [68,69] ↓ Odds of MetS (OR 0.42, 95% CI 0.21 to 0.83) (postmenopausal women) [68]. **In MASLD:** Improved lipid metabolism, IR, TG, HDL-c, ALT, HOMA-IR [76] **In obesity associated MetS:** ↑ glucose metabolism, IR and visceral fat [73] ↓ FBG, IR and BP [72] ≠WC, lipids or HbA1c [72] No benefit [71,74]	RCTs and meta-analyses in humans; heterogeneity in dose and form of vitamin D; inconsistent results; certainty of evidence for effects on triglycerides and WC rated low to very low [75]
**Choline**	Regulation of phospholipid synthesis, including serum and membrane lipid composition; is involved in membrane transport muscle structure and function, and fat and protein metabolism [162,163]	Animal-based produce like eggs, fish, beef, and milk. Choline is also abundant in beans and cruciferous vegetables [162]	Deficiencies are common in pregnancy and lactation and are associated with a vegetarian or vegan diet. ***Choline deficiency:*** ↑ risk for MASLD [164] 2.27 increased risk for CVD [165] ***Maternal deficiency*:** ↑ risk for foetal issues in neurodevelopment, the liver and metabolism [162,166]	**Prevention via adequate dietary intake:** ↓ CVD risk and all-cause mortality [167] ↓ IL-6 [168] ↓ BP and LDL-C [169] ***Safety:*** excess supplementation ↑ TMAO production with subsequent increased risk of CVD [164,170,171]	Large multicentre RCT (AHA PREDIMED) supported by small randomised cross-over trials; low GRADE meta-analysis for T2D; large cohort for TMAO
**Chromium**	Supports blood sugar regulation [85]; an essential trace mineral involved in the metabolism of carbohydrates, lipids, and proteins [84]	Whole grains, brewer’s yeast, potatoes, broccoli, seafood, Brazil nuts and hazelnuts	Deficiency is associated with characteristics of MetS ↑ FBG [89], circulating insulin, cholesterol [90], triglycerides and ↓ proportion of lean body mass [89]; [91]; inverse association between plasma chromium levels and MetS, including WC, TG, and blood glucose levels [99]; lower plasma chromium levels linked to a higher risk of T2D [94]	**In T2D patients:** **Conflicting results** ↑ glucose control in fasting and postprandial states [92] ≠FBG, weight, or BMI ↓ HOMA-IR (*p* < 0.05), indicating modest beneficial effects on insulin resistance [101] **In Coronary heart disease and T2D patients:** ↓ weight, BMI, adiposity, and lipid profiles [98] **In overweight and obese individuals:** ↓ weight, insulin and HOMA-IR [97]	Observational data consistently associate low Cr with metabolic risk; heterogeneity between trials, i.e., studied chromium form and duration; evidence supporting the idea that supplementation improves glycaemia, lipids or MetS outcomes is inconsistent
**Coenzyme Q10 (ubiquinone)**	Essential for cellular energy production via support of electron transport transfer. Functions as an antioxidant by inhibiting lipid peroxidation [172]. May improve glucose metabolism by enhancing mitochondrial function, reducing oxidative stress and influencing adiponectin levels to reduce leptin, in turn improving lipid profiles and glycaemic control [173].	Oily fish, nuts and seeds, peanuts, and pistachios	Primary deficiencies are rare. Long-term treatment with statins is associated with reduced CoQ10 levels and consequent myopathy. [174]	**Preventative:** ↓ markers of oxidative stress (CRP, MDA, and SOD) ↑ TAC ↓ inflammatory markers (IL-6, CRP, and TNF-α) [175] **In adults with MetS:** ↑ adiponectin levels ↓ inflammatory markers (IL-6, CRP, and TNF-α) [176]	Robust evidence (high-certainty GRADE) reports significantly increased adiponectin levels alongside a reduction in inflammatory markers in those supplementing with CoQ10 [176].
**Quercetin**	Activates AMP-activated protein kinase (AMPK) [86]; improves cellular energy balance and insulin sensitivity.	Berries, apples, onions, and red wine [177]	Oral bioavailability of quercetin may be low.	**On components of MetS:** ↓ FBG ↓ SBP ≠lipid markers (TG, HDL-c) ≠WC [178] **In adults with MetS:** ↓ Lipid markers (TC, LDL-c) ↓ Inflammatory marker (CRP) ≠lipid markers (TG, HDL-c) ≠inflammatory markers (IL-6, NF-α) [177]	Consistent evidence; meta-analyses of n = 16 RCTs and n = 20 RCTS with a heterogeneous sample size of 1164 adults
**Folic acid**	Supports insulin sensitivity by reducing homocysteine levels and improving endothelial function via a reduction in oxidative stress [179]	Dark-green, leafy vegetables; legumes; avocados; sunflower seeds; beetroot; and okra	An inverse association between MetS and intake and serum levels of folate [159]	**Prevention of CVD:** ≠lipid profiles; no significant impact on HDL or LDL levels [180]. **In adults with hypertension:** ↓ blood pressure [160]. **In adults with MetS:** ↓ inflammatory markers (TNF-α, IL-6 and hs-CRP) [167].	Findings moderately inconsistent; meta-analyses of RCTs: n = 45, n = 12, and n = 10
**Probiotics**	Improves gut function and intestinal permeability; supports metabolic function in antioxidant and immunomodulatory capacities [121].	Fermented products; yoghurt, kefir, sauerkraut, kimchi, tempeh, miso, kombucha, cheese, and sourdough leavened produce.	Pathological metabolic states such as MetS, obesity, and T2D are associated with gut dysbiosis [117,120]	**In adults with obesity or overweight:** ↓ weight, TC, TG [117,118] ↑ TAC [133] **In obese, postmenopausal women:** ↓ intestinal permeability, fat mass, TC, TG, blood glucose, and LPS ↓ IR [124] **The human gut:** ↑ intestinal barrier integrity [123] ↑ beneficial bacteria [126] ↓ metabolic endotoxemia [31,32] ↓ inflammation [140]	Largest evidence base in human RCTs; however, the probiotic strain and dosage vary between studies; supported by several in vivo and in vitro studies involving both animal and human cells.
**Prebiotics**	Promotes SCFA production, promotes the growth and activity of beneficial bacteria, improves gut microbiota balance and barrier function, and reduces inflammation [143,145]	Root vegetables, Jerusalem artichoke, garlic, onion, lentils, chickpeas, beans, and fermented dairy	Western dietary patterns typically high in ultra-processed foods and low in fibre, probiotic foods, and variety decrease production of small chain fatty acids (SCFA) [55]	**In healthy adults:** ↑ faecal bifidobacterial counts ↓ inflammatory markers [154] **In adults with obesity and overweight:** ↓ body weight ↓ fasting glucose ↓ insulin ↓ calorie intake ↓ ghrelin ↑ PYY [147] ↑ faecal bifidobacteria ↓ insulin, TC, TG, faecal calprotectin, CRP ↑ secretory IgA [152] **In women with T2D:** ↓ FBG, HbA1c, fasting insulin, IR ↓ CRP, TNF-α, and LPS in response to insulin [148] **Combined supplementation with probiotics:** **In prediabetic adults:** ↓ hyperglycaemia ↓ MetS [155]	Moderate evidence derived primarily from RCTs involving fewer than 50 participants; in vivo studies conducted in rats
**Lycopene**	A carotenoid with potent antioxidant activity via endothelial NO production and the inhibition of angiotensin-converting enzyme (ACE) activity and anti-inflammatory properties [181,182].	Tomatoes and tomato products (specifically, the skin), pink grapefruit, and watermelon	Lower intake is noted in obesity and in individuals consuming a typical Western diet high in processed foods low in fibre. ***Deficiency in obese individuals:*** ↑ MASLD risk [181]	**Prevention via adequate dietary intake:** ↓ SBP [182] ≠glycaemic control, obesity indices, or lipid profiles [181,182] ↑ Liver protective	Robust meta-analytic evidence synthesis of 29 RCTs for MASLD; less substantial evidence with higher heterogeneity for GC, obesity and lipid profiles; high-certainty GRADE umbrella meta-analysis for BP indices.
**Magnesium**	Reduces inflammation and improves glycaemic and insulin regulation, promoting endothelium-dependent vasodilation and normalising the lipid profile [183]	Pumpkin seeds, chia seeds, flaxseed, almonds, cashews, chickpeas, and spinach	Negative correlation between dietary magnesium intake and the prevalence of metabolic syndrome; low dietary magnesium consumption representing a potential risk factor for its development [184]	**In healthy adults:** ↑ HDL-c ↓ FBG, TG, IR, TC, BMI ↓ LDL-c with lower-dose supplementation (<300 mg/day) [185] **In adults with T2D:** ↓ SBP and DBP [185] **In adults with MetS:** ↓ SBP and DBP ↓ IL-6 ≠HbA1c, cholesterol [186] **In adults with MetS and hypomagnesaemia:** ↓ BP ↓ fasting glucose ↓ TG [187]	Moderate-to-strong evidence from two double-blind RCTs (n = 54 and n = 198), supported by a meta-analysis of 27 studies including 95,933 participants.
**Creatine**	Creatine supports cellular energy during metabolic stress, reducing oxidative stress and inflammation [188].	Red meat and fish	May improve body composition, particularly when combined with resistance training. However, evidence for effects on glucose metabolism and insulin sensitivity remain inconsistent.	**In adults:** ≠FBG (older adults) [189] ≠fasting insulin, HOMA-IR, inflammatory markers [189] ≠glucose tolerance, insulin sensitivity [190] **In adults ≥50 + resistance training**: ↑ lean tissue mass ↓ body fat % and fat mass [191] **In adults with T2D:** ↓ FBG [192] ↓ glycaemia during meal tolerance test [193] ↓ HbA1c [193] ≠HbA1c, insulin, C-peptide [192]	Limited evidence. Supported by a meta-analysis of 19 studies (n = 609) and a few small clinical studies. Overall, findings were inconsistent, and studies were generally of short duration.
**Polyphenols**	Antioxidant and anti-inflammatory influences on gut microbiota and vascular function via reduced oxidative stress and improved glucose and lipid metabolism [194,195].	Vegetables, berries, grapes (specifically, skins). herbs, nuts, seeds, legumes, tea, coffee and cocoa	Lower intake likely in individuals consuming a typical Western diet high in processed foods and low in variety and fibre.	***Resveratrol*** **In adults with T2D:** ↓ moderate for SBP, DBP, MAP, PP, HOMA-IR, FBG ≠Fat Mass Clinically important improvements on HbA1C levels [196] **In adults with T2D and obesity:** Improvements in WC, HbA1C, LDL-c, TC, HDL-c [197]. **In adults with MetS:** ↓ FBG, WC, TG ≠TC [198] ***Curcumin*** **In adults with MetS:** Improvements in WC, FBS, DPB, HDL-c, TNF-α, CRP, and MDA [199]	Robust umbrella review of meta-analyses of RCTs (HbA1c), meta-analysis of 25 RCTs (WC, HbA1c, and lipids), and meta-analysis of 10 RCTs (FBG); curcumin evidence is limited; meta-analysis of 13 RCTs reports high heterogeneity; very low to moderate GRADE scores; limited ethnic diversity.

Symbols indicate: ↓ reduction; ↑ improvement; ≠ no change.

**Table 2:** Comparison of nutraceutical regulatory frameworks.

	European Union	United States	United Kingdom
**Classification**	Food supplements	Dietary supplements (food)	Food supplements
**Pre-market approval**	Required for novel ingredients and health claims	Generally not required	Limited, broadly similar to EU framework
**Safety responsibility**	Shared between manufacturers and regulators (EFSA)	Manufacturer-led	National regulatory authorities
**Regulatory approach**	Precautionary	Predominantly post-market surveillance	Hybrid approach

## Data Availability

No new data were created or analysed in this study. Data sharing is not applicable to this article.
